# Combined effects of increased temperature and endocrine disrupting pollutants on sex determination, survival, and development across generations

**DOI:** 10.1038/s41598-017-09631-1

**Published:** 2017-08-24

**Authors:** Bethany M. DeCourten, Susanne M. Brander

**Affiliations:** 1 Department of Biology and Marine Biology, University of North Carolina Wilmington, 601 S. College Road, Wilmington, NC 28403 USA; 2 Department of Environmental and Molecular Toxicology, Oregon State University, 1007 Agricultural and Life Sciences Building, Corvallis, OR 97331 USA

## Abstract

Understanding the combined effects of anthropogenic impacts such as climate change and pollution on aquatic ecosystems is critical. However, little is known about how predicted temperature increases may affect the activity of endocrine disrupting compounds (EDCs), particularly in species with plasticity in sex determination. We investigated the effects of a concomitant increase in temperature and exposure to estrogenic EDCs on reproduction and development in an estuarine model organism *(Menidia beryllina)* across multiple generations. Parents (P) were exposed to environmental levels of the estrogenic insecticide bifenthrin or ethinylestradiol (EE2) at 22 °C and 28 °C for 14 days prior to the initiation of spawning trials. Embryos in the F1 generation were exposed to EDCs until 21 days post hatch (dph), reared to adulthood in clean water at elevated temperatures, and spawned. F1 sex ratios were significantly influenced by elevated temperature and EDCs, potentially altering adaptive development. We also observed fewer viable offspring and increased developmental deformities in the F1 and F2 generations, with a greater impact on F2 juveniles. These findings enhance our understanding of responses to EDCs in the context of climate change and may demonstrate heritable effects. Our study represents the first multigenerational assessment of elevated temperatures in combination with environmentally relevant concentrations of commonly detected endocrine disruptors in a model vertebrate species.

## Introduction

Plasticity in sexual determination, often in response to environmental factors, is governed by a set of mechanisms that adjust sex ratio without altering genotype, and has evolved multiple times in poikilothermic taxa such as reptiles, amphibians and fish. For example, some organisms differentiate based on environmental cues, such as temperature^1^. This can occur when seasonal differences in development between the sexes optimizes adult fecundity, in some cases, allowing females to grow larger than males^2–4^. In stable populations, these skewed sex ratios typically average to a 1:1 ratio over time^5, 6^, however little is known about how organisms with this sensitive physiology may be influenced by anthropogenic stressors. For example, the increases in temperature associated with global climate change^7^ can lead to the disruption of reproductive outcomes in organisms with plasticity in sex determination^8–10^. Fish species that tend to produce male-biased cohorts at higher temperatures, such as zebrafish and silversides, may also experience population decline with increasing temperature^11^, as the reproductive capacity of the population decreases. Since population growth is largely determined by female fecundity, increases in temperatures that cause a skew towards a male bias could lead to impaired populations under projected climate change scenarios^12, 13^. Other environmental stressors, such as exposure to endocrine disrupting compounds (EDCs), can alter the sex ratio of organisms that have plasticity in sex determination^14^. EDCs are known to interfere with hormone signaling across taxa^15^, and elevated temperatures appear to increase the severity of EDC effects^16^. Given that exposure to pollutants occurs within the context of global climate change, it is essential that we understand the combined implications in species with sensitive physiology, especially those inhabiting highly impacted areas such as estuaries^17^. Understanding their collective effect(s) is of particular concern since both rising temperatures and endocrine disrupting compounds (EDCs) have been separately linked to altered growth, decreased sperm motility, impaired embryo development, reduced fecundity, altered behavior, and decreased survival of fish at early, more sensitive, life stages^18–20^.

The EDCs bifenthrin (BF, pyrethroid pesticide) and ethinylestradiol (EE2, oral contraceptive) are commonly used in developed countries and frequently contaminate watersheds^21, 22^. Both compounds alter estrogen signaling in fishes at environmentally relevant concentrations^15^. Bifenthrin has been shown to impair development and to reduce reproductive success in fish by influencing hormone levels, reproductive behavior and development of gametes, decreasing the gonadal-somatic-index (GSI) in fish and hindering the development of healthy ovarian follicles^16, 18, 23, 24^. Because the metabolite of bifenthrin is a more potent estrogenic endocrine disruptor than the parent compound^25^ we expect that elevated temperature will influence its toxicity as metabolic rates increase with temperature^26^. The well-studied synthetic estrogen, EE2, has been shown to affect behavior, reproduction, and development^9, 14, 22, 27, 28^. As a potent estrogen, EE2 has been used in other studies as a positive control when testing for the estrogenic effects of other compounds^29, 30^.

The inland silverside (*Menidia beryllina*) is a widespread estuarine species of ecological importance with a short generation time (6–8 months)^23, 31^. *M. beryllina*, which is thought to exhibit plasticity in sex determination, as other *Menidia* species do^32^, is also highly sensitive to EDCs^14, 18, 33^. Our objective was to assess how exposure to commonly detected EDCs (EE2, BF) at environmentally relevant levels (1 ng/L)^22, 34^ affects sex ratio and reproductive success in an organism with said plasticity. Since multigenerational studies are important for determining long-term effects of stressors in wild populations^35^, we examined the relationship between elevated temperature (F0, F1, F2) and EDCs (F0, F1) in *M. beryllina* over three generations.

The critical temperature necessary to maintain a female-biased sex ratio in other *Menidia* species closely related to *M. beryllina*, which have a temperature sensitive period continuing until roughly 21 days post hatch (dph), is ≤ 25 °C, above which the population begins to skew toward a male bias^2^. We chose to test a temperature near the upper thermal limit at which *M. beryllina* are observed spawning in the wild (28 °C) to ensure spawning success^31^ and compared this to an ambient temperature of 22 °C. The 28 °C temperature represents the worst-case scenario of mean global temperature increases modeled by the more extreme (RCP 8.5) models put forth by the Intergovernmental Panel for Climate Change^7^. As such, we exposed adult fish (F0) to EDCs at 22 °C and 28 °C for 14 days prior to spawning. The F1 generation was exposed from gamete to larval stages (to 21 dph), then reared to adulthood in clean water at the experimental temperatures. This period of approximately 35 days is relevant to that of a pulse exposure in the wild^36^. The F2 generation did not undergo any chemical exposure, to test for the heritability of the observed effects (Supplemental Fig. 1). We hypothesized that EDCs would reduce egg production and offspring production, with stronger effects measured at higher temperatures, and that sex ratios would be influenced by estrogenic EDC (BF or EE2) exposure. We hypothesized that the F2 generation, only exposed indirectly as germ cells to EDCs, would exhibit effects similar to those of generations exposed to both a warmer temperature and EDCs. To the best of our knowledge, this is the first study to combine an assessment of environmentally relevant stressors in the context of climate change over multiple generations in a model vertebrate species.

## Results and Discussion

**Figure 1:**
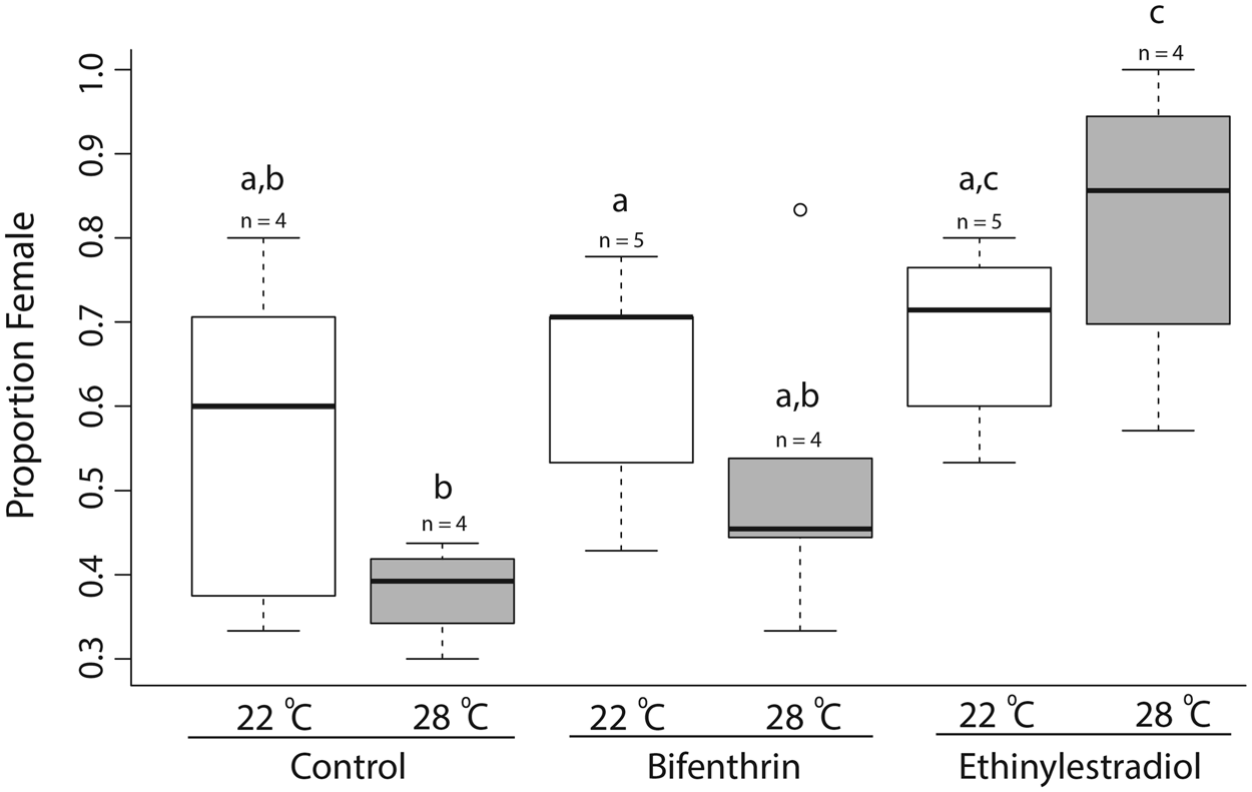
Box plot of the proportion of females in the F1 generation by treatment, demonstrating plasticity in sexual determination in *M. beryllina*. Boxes denote the inter-quartile range and whiskers represent the upper and lower quartiles, with median denoted solid bars. Treatments that share the same lowercase letters are not significantly different (p < 0.05). Lowercase “n” represents the number of replicate tanks in each treatment.

Our study demonstrated that sex ratios of *M. beryllina* can be influenced by abiotic stressors, similar to that of their congeners (binomial GLM, n = 26, p = 0.01215, Fig. 1)^1, 32^. We did not detect any significant differences in sex ratio among EDC treatments at the ambient temperature (22 °C). However, the proportion of females in the warm EE2 (28 °C) treatment was significantly greater than that in the warm and ambient controls (binomial GLM followed by Tukey HSD, n = 4 per treatment, p = 5.7 × 10^-5^, p = 0.0144, respectively; Fig. 1). We also found that the sex ratios in the 28 °C BF and EE2 significantly differed (binomial GLM followed by Tukey HSD, n = 4 per treatment, p = 0.0116, Fig. 1), confirming that EE2 acts as a more potent estrogen. Although the difference between controls was not significant at the 0.05 alpha level, the low p-value (0.0588) (binomial GLM followed by Tukey HSD, n = 4 per treatment,), suggests that this species may adhere to the expected pattern of a male biased sex ratio at warmer temperatures^1^ (Fig. 1). The difference in sex ratio between temperatures observed in the EE2 treatments, although not significant (binomial GLM followed by Tukey HSD, n = 4–5 per treatment, p = 0.0703) contradicts the expected influence of temperature on sex ratio based on studies with closely related species, in which the sex ratio is expected to skew toward a male bias at elevated temperatures^1^ (Fig. 1). Our findings suggest that estrogenic EDCs may be able to inhibit or reverse the expected sexual differentiation at high temperatures in fish with sexual plasticity. A recent study demonstrated a similar response in zebrafish, with exposure to estrogens inhibiting masculinization of fish at higher temperatures^9^. Another study showed that development can be influenced by thermal fluctuations in fish, in some cases causing sex reversal in the fathead minnow^37^. Our data support these findings and suggest that both elevated temperatures and exposure to estrogenic EDCs can disrupt sexual differentiation in fish. Plasticity in sex determination is favored when a temporal difference between production of male and female offspring increases fitness of the population^1^. Our results indicate that presence of EDCs may counteract the adaptive advantage of plastic sex determination, potentially increasing the risk of population collapse.

**Figure 2:**
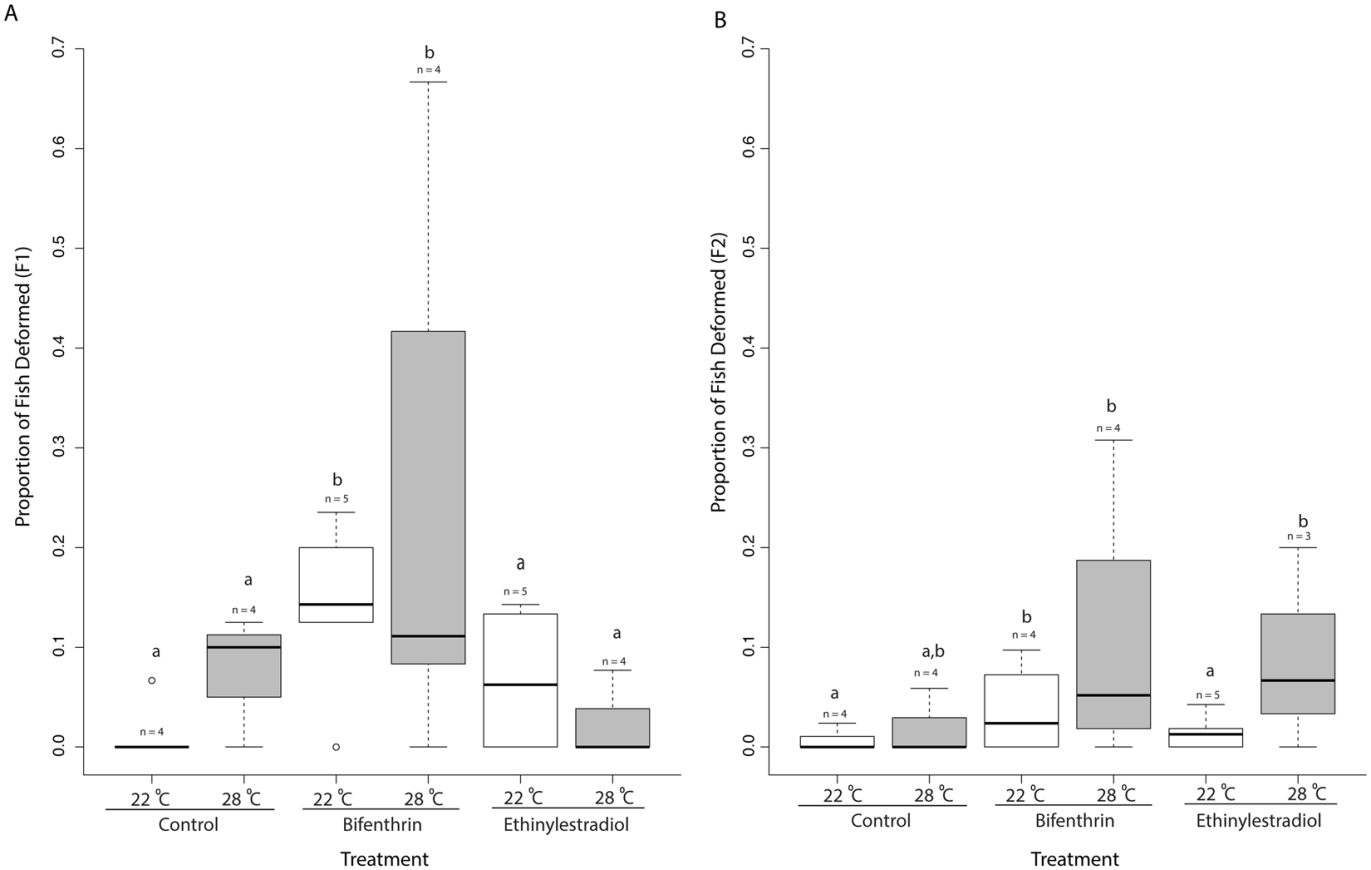
Box plot of the proportion of F1 adults and F2 larvae exhibiting developmental deformities. Boxes denote the inter-quartile range and whiskers represent the upper and lower quartiles, with median denoted solid bars Treatments that share the same lowercase letters are not significantly different (p < 0.05). Lowercase “n” represents the number of replicate tanks in each treatment.

Developmental deformities were observed throughout the rearing of the F1 and F2 generations. Deformed fish were smaller and darker than healthy fish, and exhibited a varying degree of caudal fin deformities (Supplemental Image 1). There were threefold more deformed F1 adults (Fig. 2A) collectively in BF treatments than in controls and EE2 (binomial GLM followed by Tukey HSD, n = 4–5 per treatment, p = 0.0042, Fig. 2) treatments, with no effect of temperature observed. Because the F2 generation was not reared to adulthood, deformities were assessed during the 21 dph sampling. Presence of deformities in the unexposed F2 generations show that these effects can be transferred to subsequent generations either due to indirect exposure of primordial germ cells (contained within F1 parents) or epigenetically. There was a higher proportion of deformed F2 larvae (Fig. 2B) in the 22 °C BF, 28 °C BF and 28 °C EE2 treatments compared to the 22 °C control indicating that the full effect of EDCs on development may not be realized in a single generation (binomial GLM, followed by Tukey HSD, n=4–5 per treatment, p = 0.00787, p = 0.00329, p = 0.0038, respectively, Fig. 2B). There were six-fold more deformed larvae in the 28 °C EE2 (n = 4 per treatment) than 22 °C EE2 (binomial GLM followed by Tukey HSD, n = 5 per treatment, p = 0.00576, Fig. 2B) treatments, showing that temperature can exacerbate the development of deformities following parental EDC exposure. Estrogenic chemicals have been shown to cause developmental abnormalities and to impair locomotor function in fish exposed to the chemical as embryos^16^. Deformities in this study following EDC exposure resulted in impaired caudal development and swimming capabilities which could have a strong effect on feeding success and predator avoidance in a natural habitat^38^. In the F2 generation, there was an effect of both EDCs, with EE2 effects only apparent at higher temperature, indicating that the response is influenced by temperature. It was previously demonstrated that deformities following parental exposure to EDCs can have heritable effects on morphological deformities in subsequent generations^39^. Our results suggest that deformities may not only be heritable but may worsen in subsequent generations. These data do not indicate whether populations will be able to adapt to chronic exposure to anthropogenic stressors.

**Figure 3:**
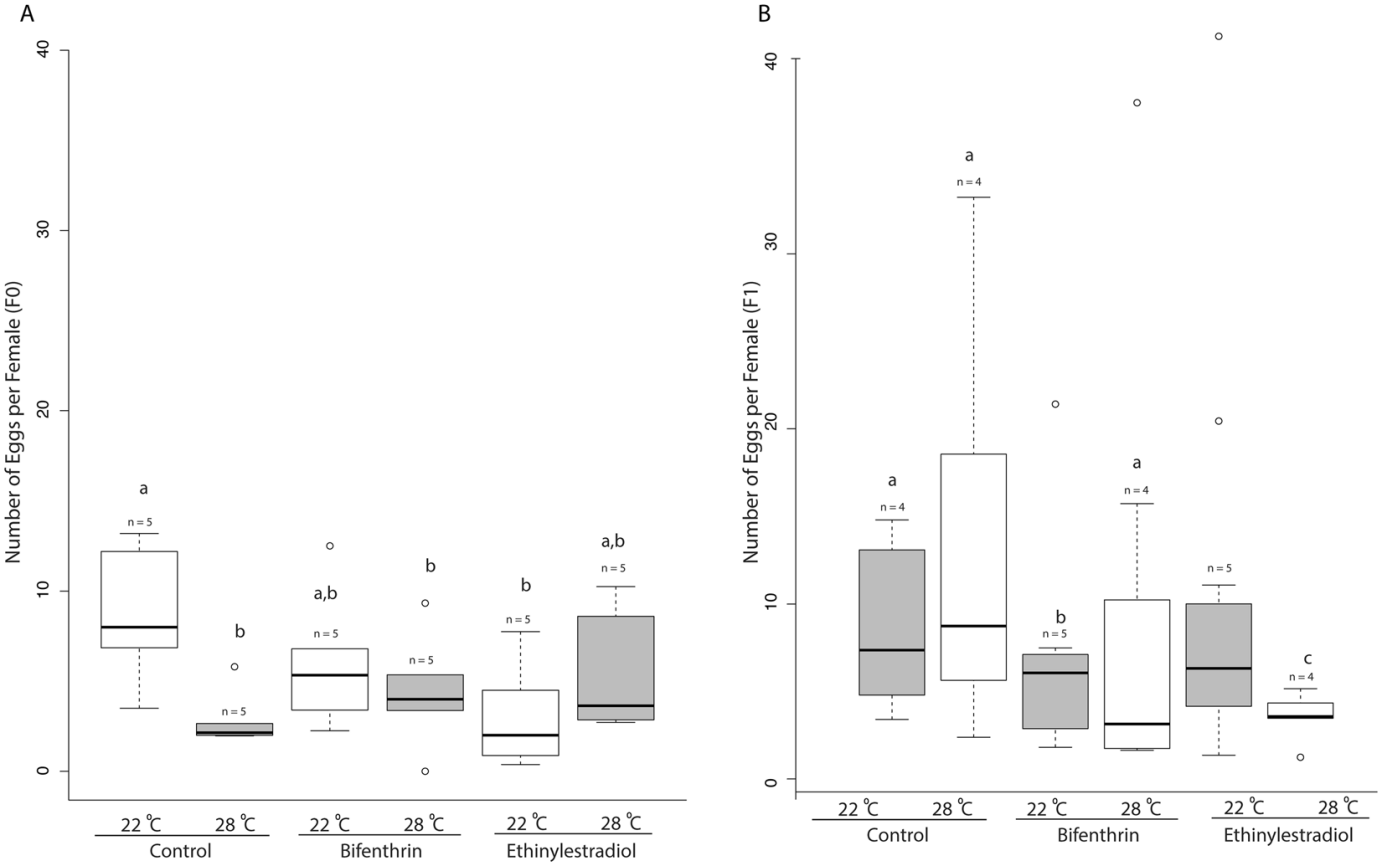
Box plot of the eggs produced in spawning trials (per female) in the F0 generation (**A**) and F1 generations (**B**). Boxes denote the inter-quartile range and whiskers represent the upper and lower quartiles, with median denoted solid bars. Treatments that share the same lowercase letters are not significantly different (p < 0.05). Lowercase “n” represents the number of replicate groups in each treatment.

We found that an increase in temperature (to 28 °C) reduced the average number of eggs produced per female (Fig. 3A) in the F0 generation control groups and (Poisson GLM followed by Tukey HSD, n = 5 per replicate, p = 0.00239). When compared to the 22 °C control we found that EE2 and BF significantly reduced the number of eggs in 22 °C and 28 °C respectively (Poisson GLM followed by Tukey HSD, n = 5 per treatment, p = 0.00239). These results suggest that BF acts as a more potent estrogen at higher temperatures, likely due to higher rates of conversion into the more potent metabolite^25^. Conversely, the opposite was found in the EE2 treatments suggesting that this chemical is less potent at higher temperatures. These effects differed from the effects observed in the F1 generation (Fig. 3B) where we found a reduction in egg production following exposure to BF (22 °C, Poisson GLM followed by Tukey HSD, n = 5 per treatment, p = 0.00119, Fig. 3B), and the largest reduction of egg production in the EE2 (28 °C, Poisson GLM followed by Tukey HSD, n = 4 per treatment, p = 1.06 × 10^−5^, Fig. 3B) when compared to the controls. These data confirm that EE2 is a highly potent EDC capable of causing reduced fecundity as seen in previous studies^40, 41^. We detected differences between the 22 °C and 28 °C groups for both BF (Poisson GLM followed by Tukey HSD, n = 4 per treatment, p = 0.0175) and EE2 (Poisson GLM followed by Tukey HSD, n = 4 per treatment, p = 4.81 × 10^−5^) (Fig. 3). The fecundity of *M. beryllina* slows as temperatures approach the critical limit of 31 °C, after which spawning ceases^31^. Separately, it has been shown that both low levels of estrogenic compounds and elevated temperatures can decrease egg production in fish^18, 40, 42^, however literature is lacking on the potential interactions of these stressors and the effects on fecundity. Our results indicate that when combining these stressors temperature can influence the effects of EDCs on egg production, as we did not detect differences between temperatures in the control groups. In accordance with the results from the spawning assays, we also found that female gonadal somatic index (GSI) (F0, F1) decreased (Supplemental Fig. 2) following temperature increases and EDC exposure (two-way ANOVA, n = 4–5, p < 0.03). Previous studies with estrogenic compounds have shown that these EDCs can reduce gonadal size, thus decreasing reproductive capacity^15, 33, 42^. Our results support these findings and expand upon the possible interaction of elevated temperature and EDC exposure.

**Figure 4:**
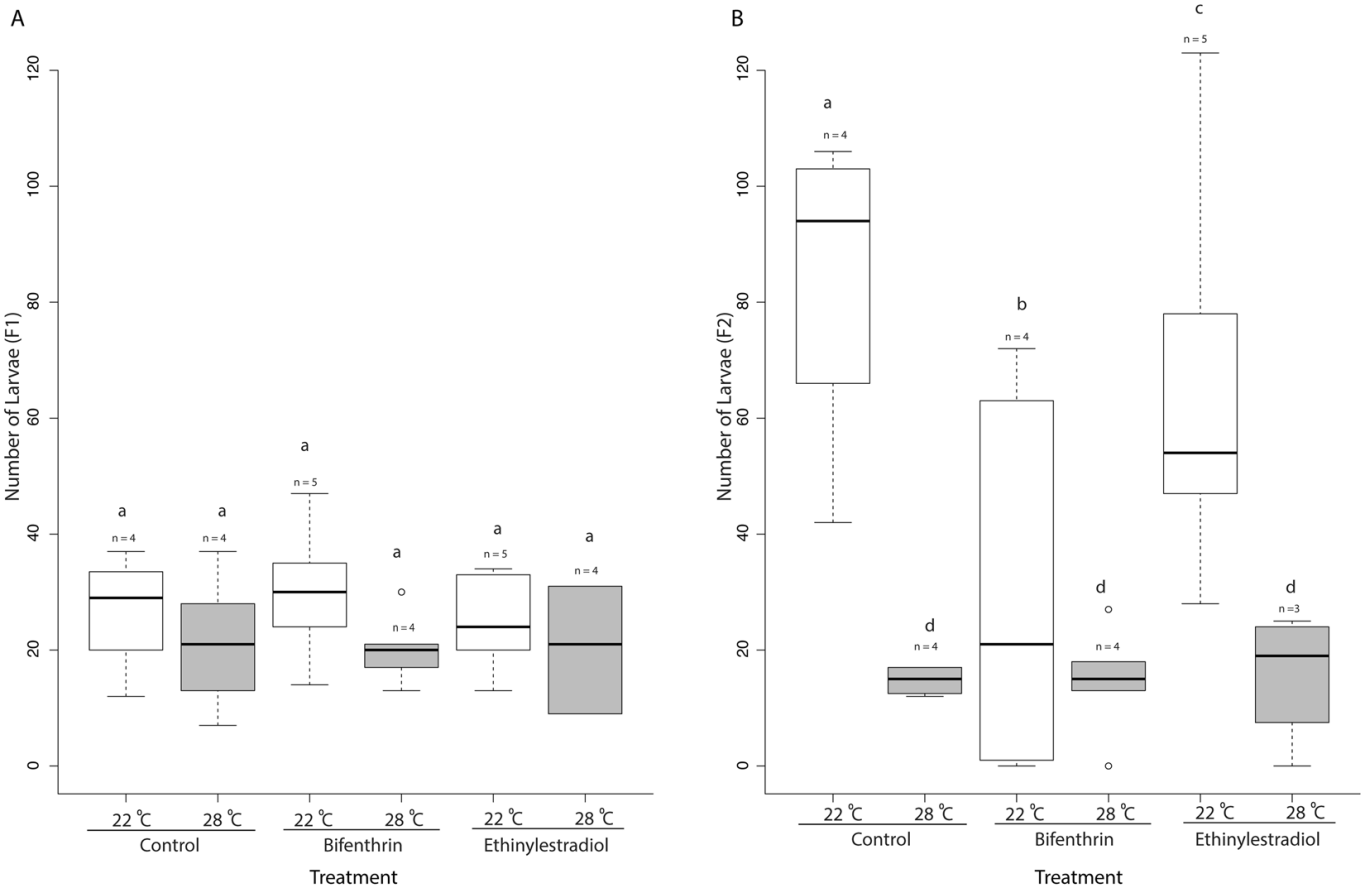
Box plot of the number of larvae assessed at 21 dph produced in the F1 generation and F2 generations. Boxes denote the inter-quartile range and whiskers represent the upper and lower quartiles, with median denoted solid bars. Treatments that share the same lowercase letters are not significantly different (p < 0.05). Lowercase “n” represents the number of replicate tanks in each treatment.

Larval production (assessed at 21 dph, incorporating both hatch and early survival) decreased in all 28 °C treatments (F2) when compared to the 22 °C control (two-way ANOVA followed by Tukey HSD, n = 4 per treatment (EE2 and MeOH) n = 5 (BF), p < 2.00 × 10^−16^ Fig. 4B). Within the 22 °C treatments the fewest larvae were produced in the BF treatments (two-way ANOVA followed by Tukey HSD, n = 5 per treatment, p < 3.41 × 10^−14^, Fig. 4B) followed by the EE2 treatments (two-way ANOVA followed by Tukey HSD, n = 4 per treatment, p = 0.00373; Fig. 4B). Offspring quantities in the F2 generation were affected by both EDC exposure and temperature increases with a greater reduction in larvae than the previous generation, as there were no significant differences between treatments in the F1 generation (Fig. 4A). These results indicate that the severity of these effects may not be realized within a single generation as previously observed with estrogenic EDCS^43^. Additionally, this suggests that offspring response to EDCs is dependent on the timing of parental exposure. F1 larvae were produced following parental exposure at the adult stage, as opposed to the F2 larvae which were produced following parental exposure at the larval stage. These results support the idea that exposure to EDCs at early life stages may have stronger heritable effects than exposures at adult stages^44^. This study suggests that increasing temperatures associated with climate change may negatively impact the reproductive output and viability of offspring of *Menidia* species in North America and Atherinid species in general, which are found in numerous temperate zones globally. Temperate fish exposed continuously to higher temperatures without seasonal winter cooling produce lower quality offspring due to reduced size and nutritional quality of the eggs produced^45^, possibly reducing offspring viability. Furthermore, offspring production in fish exposed indirectly as primordial germ cells, as our F2 generation was, can also be reduced^41^. This supports the idea that populations exposed to EDCs may not be able to adapt to estrogenic EDC exposure as temperatures increase, with deleterious effects persisting throughout generations^40^. To confirm this, future assessments should be conducted at least through the F3 or ideally through the F4 generation.

In closely related *Menidia* species, producing more females at colder temperatures (representative of early spawning months) allows them to mature earlier, grow larger (fecundity increases with female size), and produce more eggs over the entirety of the spawning season^32^. Results from this study suggest that combined exposure to elevated temperature and/or EDCs alters sex ratios, reduces offspring number, and hinders their development. Some effects of these stressors were shown to impact subsequent generations, suggesting that anthropogenic stressors can cause persistent population level effects. The implications of this study extend to other vertebrates with plasticity in sex determination, as members of many taxa (i.e., reptiles and amphibians) exhibit similar responses to temperature change and EDCs. For example, in some reptiles elevated temperature can lead to a female-biased sex ratio, as opposed to a male-biased sex ratio as in *Menidia* species, and can also affect the development of offspring^3^, as we observed here. In the face of global climate change, silversides and other similarly adapted species may experience greater population declines due to reduced population-level fecundity and a decrease in viable offspring. It has been shown that temperature increases expected under current climate change projections will lead to population collapse, possibly within the next 50 years, particularly for species with temperature-dependent plasticity in sexual development in populations that are already threatened^46, 47^. Our study demonstrates that the risk of collapse may be exacerbated in contaminated waterways, even at relatively low concentrations of chemicals (1 ng/L), possibly leading to more rapid localized extinction events. Our results also imply that some of the effects of temperature on reproduction, such as decreased larval production, are measureable in subsequent generations, especially when combined with exposure to endocrine disruptors. Additional research is necessary to determine the potential additive or synergistic effects of multiple anthropogenic stressors across generations, within a changing climate, to understand the potential for long-term population-level effects.

## Methods

### Rearing of parental fish

Fish in the parental generation (P) were obtained from Aquatic Biosystems (Fort Collins, CO) at 30 days post hatch (dph) and reared to adulthood in 25 °C, 15 ppt water at the University of North Carolina, Wilmington’s Center for Marine Science. Fish were fed a diet of Hikari tropical micro pellets (Kyorin Food Industries, Ltd, Japan) and live *Artemia* nauplii supplemented with Selcon™ (American Marine Inc., Ridgefield, CT), during rearing and experimental periods. Adult fish (341 dph) were transported to experimental chambers and acclimated to either 22 °C or 28 °C for 3 weeks prior to experimental exposures under a continuous 16:8 light-dark cycle. Fish in the F0 generation were separated into 5 replicate jars containing 10–13 for each treatment and temperature used in the study.

### EDC exposures

Parental exposures were initiated by replacing approximately 90 percent of the water in experimental tanks with water containing the target concentration of chemicals used in the study. For the control groups, 1 mL/L of methanol was added to control for any effects that could be attributed to the solvent, inbreeding depression, or experimental heat and handling stress. BF and EE2 treatments contained 1 ng/L of bifenthrin (purity: 99.5%, Chem Service, West Chester, PN) or 1ng/L 17α-ethinylestradiol (EE2) (purity: ≥98%, Sigma-Aldrich, St. Louis, MO), respectively, suspended in 1 mL/L of methanol due to the hydrophobic nature of the EDCs. Adult fish (F0, 248 dph) were exposed for 14 days in 7.6 L glass jars, containing approximately 6 L of artificial seawater. F1 embryos were produced during the parental exposure and continued until all larvae had reached 21 dph, approximately the time of sexual determination. Exposure jars were placed in 60 L water baths using aquarium heaters to maintain temperature (21.9 ± S.D. 0.41 and 27.7 ± S.D. 0.89, for temperature treatments respectively). Fish were maintained at a constant salinity target of 15 ppt optimal for growth and survival in *M. beryllina* ^36^. Artificial seawater (15 ppt) was made daily by adding Instant Ocean to distilled water and aerated for 24 hours prior to use. Solutions with experimental chemicals were mixed in glass containers, immediately prior to daily water changes. Jars were continuously aerated and water quality parameters including temperature (21.9 ± S.D. 0.41 and 27.7 ± S.D. 0.89, for temperature treatments respectively), dissolved oxygen (5.81 ± S.D. 0.98), pH (7.94 ± S.D. 0.56), salinity (16.26 ± S.D. 1.59), and ammonia (0.22 ± S.D. 0.76) were measured with a YSI Professional Plus Quatro water quality meter daily before and after water changes in two representative control jars at each temperature. Temperature of water baths was measured once daily for the duration of the experiment. Sixty percent of the water was removed and replaced daily following thorough removal of debris in experimental chambers. All air pumps and heaters were connected to an emergency power source to ensure continuous function. Adult fish were fed a diet of Hikari fish food pellets, freeze-dried blood worms and live *Artemia* nauplii supplemented with Selcon^TM^ (vitamin supplement). Larvae were fed live rotifers (*Brachionus rotundiformis*) from 0–14 dph, and a combination of live *Artemia* and ground Hikari pellets from 10 dph to adulthood. Fish were fed daily and allowed to feed for one hour prior to water change. Following exposures, spawning trials were initiated with the parental generation adults, and F1 larvae were transferred to clean tanks and reared to adulthood at experimental temperatures.

### Analytical Chemistry

Water samples to control for methods were mixed using experimental methods and materials in 950 mL sterile amber bottles, transported on wet ice and stored in the dark at 4 °C for no longer than 24 hours. Extraction was performed using conditioned 6 mL solid phase-extraction C_18_ cartridges (Superclean^TM^ 500 mg, Sigma- Aldrich) at a slow drip. Columns were rinsed twice with 5 mL of a 1:1 solution of hexane:ethyl acetate to elute the bifenthrin. The solvent elution (10 mL) was concentrated to 0.4 mL under a gentle stream of nitrogen. Final extract was analyzed using gas chromatography negative chemical ionization mass spectrometry (GC-NCI-MS) on an Agilent 5973 series gas chromatograph (Agilent Technologies, Palo Alto, CA) equipped with a split-splitless injector (280 °C, splitless, 1.5 min purge time). The columns used were Supelco DB- 5MS column (30 m × 0.25 mm with a 0.3 μm film thickness). The instrument was calibrated using nine sets of standard bifenthrin solutions, the surrogate trans-permethrin D6 (EQ Laboratories, Atlanta, GA), and the internal standard dibromooctafluorobiphenyl (Chem Service, West Chester, PA) in hexane. Controls were conducted by analyzing a method blank of deionized water (Milli-Q) to ensure samples were not contaminated. The Surrogate was added to each sample before extraction to monitor matrix effects and method performance. The detection limit of bifenthrin in whole water was 0.6 ng/L. No bifenthrin was detected in the controls or method blank. Measured concentrations of bifenthrin were 1.3 ng/L. Surrogate recovery ranged from 133.84%, with corrected value of bifenthrin concentration being 0.89 ng/L. Analytical chemistry was not performed on EE2 treatment water, but those solutions were made according to previously established protocols^18, 25^. All stock and experimental solutions were mixed in the same manner as bifenthrin to ensure like concentrations.

### Spawning assay

On day 14 of parental exposures, a single spawning trial including 8–15 adult fish (depending on exposure mortality) for the parental generation and 9–18 fish for the F1 generation, was initiated by the addition of spawning substrate (6 inch strands of bleached and rinsed dye free yarn), as described in Brander *et al*.^18^. Substrate remained in the tanks for 24 h before removal. The spawning trial with adults from the F1 generation were initiated at time of maturity, to test the effects of larval exposure only. F1 spawning trials were extended to 48 hours to increase the likelihood of spawning. Fertilized and unfertilized eggs were counted under a dissecting microscope to assess reproductive output. After a 24 h recovery period following the spawning trial, new spawning substrate was added to each jar and fish were allowed to spawn for 48 h to produce the embryos of the subsequent generation. After the spawning assays, all adult fish were euthanized in an overdose solution of MS-222 and immediately weighed, measured and dissected to determine sex and gonadal somatic index (GSI).

### Larval rearing

Spawning substrate from the second spawning event remained in experimental jars allowing attached embryos to hatch without transfer. All F1 larvae were reared with chemical exposure until 21 dph at which point a subset of 5 larvae were sampled at 21 dph, weighed and measured to obtain morphometric data and preserved to be used for later molecular analysis and all remaining fish were transferred to clean 37.5 L round black polypropylene tanks to be reared to sexual maturity at either 22 °C or 28 °C (21.9 ± S.D. 0.41 and 27.7 ± S.D. 0.89, for temperature treatments respectively). Each tank was equipped with Marineland Penguin PF0100B filter, one Eheim Jager aquarium thermostat heater (75 watt) and Resun AF-2009D automatic feeder. F2 larvae were spawned and reared in clean water at experimental temperatures until 21 dph, to test the effect of parental exposure on offspring morphology and viability. At this time time, all larvae were sampled and the experiment was terminated. All experiments were conducted in accordance with UNCW Institutional Animal Care and Use Committee (IACUC) protocol #A1314-010 and #A1415-010. These protocols included methods for all experiments involving live animals, and were approved by UNCW IACUC prior to experimentation.

### Statistical analysis

We tested for significant differences with each endpoint using generalized linear models, followed by Tukey’s post hoc comparisons (GLM) Morphometric data (SL and mas) obtained from all fish used in spawning trials (9–18 per replicate), a subset of larvae (5 per replicate, 21 dph) in the F1 generation and all larvae produced (maximum of 50) in the F2 generation (21 dph). We tested for differences in morphometric data using an ANCOVA (n = 5 and n = 4 for the parental and F1 generations respectively) followed by Tukey’s post-hoc comparison. For all post-hoc comparisons, p-values were calculated in a manner that preserved a 0.05 false discovery rate^48, 49^. We tested for differences in egg production by standardizing the number of eggs produced, by the number of females in each jar before using a generalized linear model (log link, Poisson error distribution), followed by Tukey’s post hoc comparisons. Differences in sex ratios of fish used in the F1 spawning trial (10–15 per replicate) were tested using a generalized linear model (binomial distribution) followed by Tukey’s post hoc comparisons comparison. We tested for differences in the proportion of deformed fish observed at the time of sampling in the F1 (10–15 per replicate) and F2 (all remaining fish in each replicate) generations using a binomial generalized linear model and Tukey’s post hoc comparison. For further information on the number of fish in each replicate please refer to Supplemental Table 2. All statistical analyses were performed in R (v 3.3.1); post-hoc comparisons were made using package multcomp^49^. The datasets generated during and/or analysed during the current study are available from the corresponding author on reasonable request.

## Electronic supplementary material

Supplementary Information
