# Supplementary material for: Combined effects of increased temperature and endocrine disrupting pollutants on sex determination, survival, and development across generations
Source: Sci Rep. 2017 Aug 24;7:9310. doi: 10.1038/s41598-017-09631-1 (PMC5571109; doi:10.1038/s41598-017-09631-1)
Supplement: Supplementary file 1 — Supplementary Information [file 41598_2017_9631_MOESM1_ESM.doc]

**Supplementary information for**

Combined effects of increased temperature and endocrine disrupting pollutants on sex determination, survival, and development across generations

DeCourten, Bethany M.^1*^, and Brander, Susanne M.^1^

1. Department of Biology and Marine Biology, University of North Carolina, Wilmington. 601 S. College Road, Wilmington, NC, 28403

*Corresponding author: bmd5520@uncw.edu

**One single PDF contains:**

*Supplemental figures*

Supplemental figures 1and 2

*Supplemental Tables*

Supplemental Tables 1and 2

*Supplemental Image 1*

**Supplemental Figure 1**. A diagram of experimental design. Shaded boxes denote periods of EDC exposure.


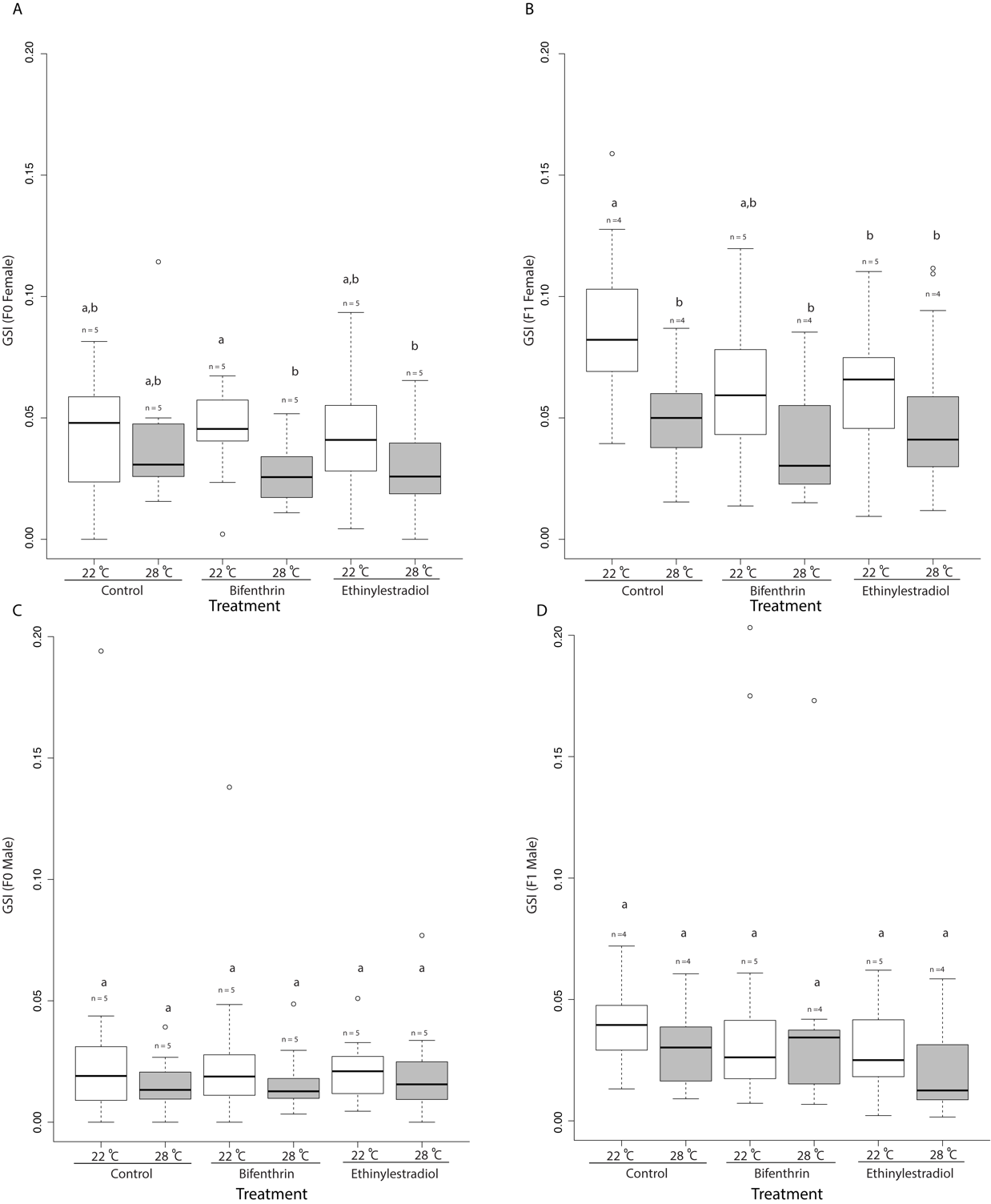


**Supplemental Figure 2**. Box plot of the number of the GSI of F0 females (A), F1 females (B)), F0 males (C), F1 males (D). Boxes denote the inter-quartile range and whiskers represent the upper and lower quartiles, with median denoted solid bars. Treatments that share lowercase letters are not significantly different (p<0.05). Lowercase “n” represents the number of replicate tanks in each group.

**Supplemental Table 2.** The number of fish in each replicate at different life stages for each replicate used in the experiment

**Supplemental image 1.** Adult silversides with caud
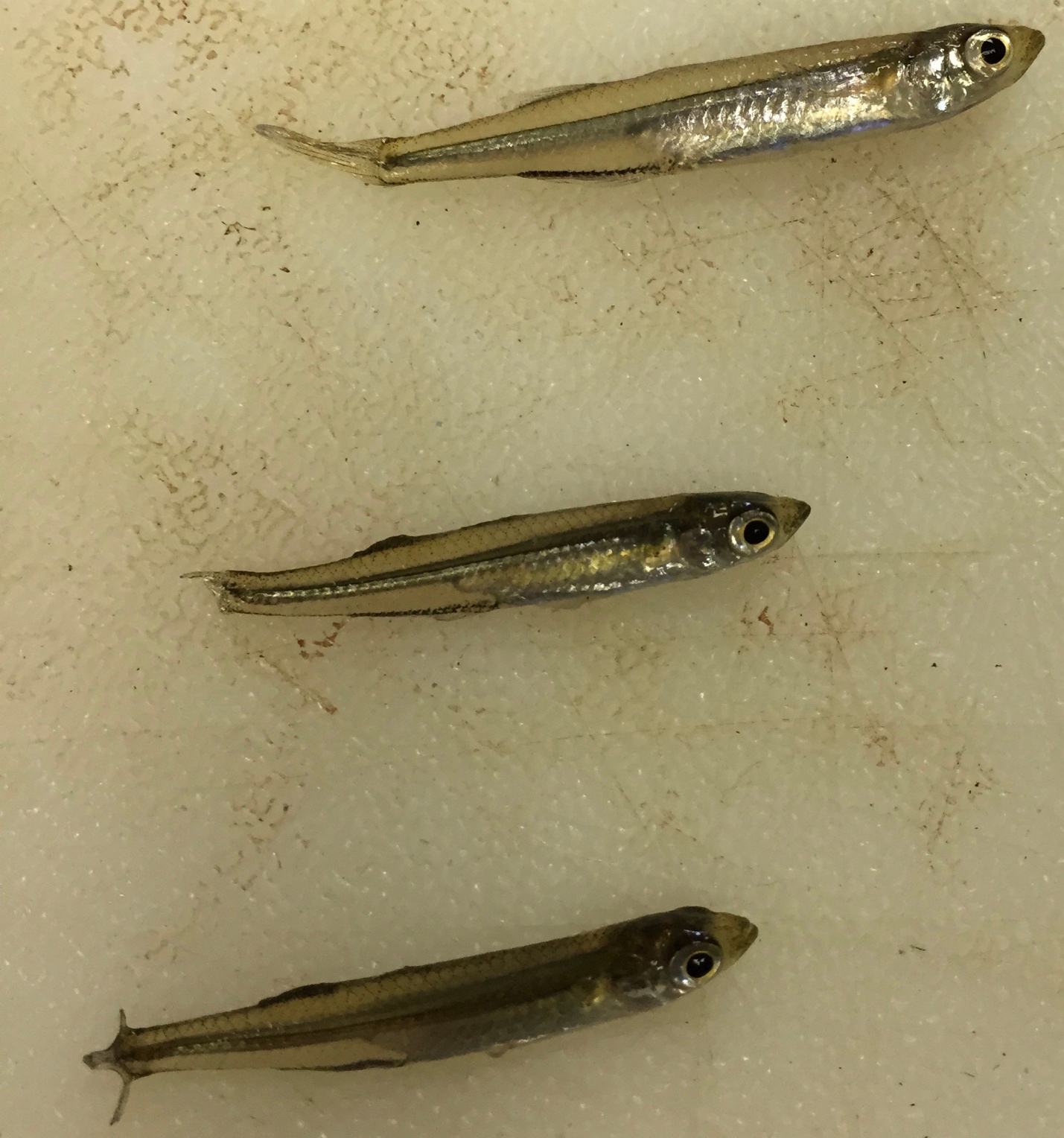
al deformations.
